# Supplementary material for: The “Self” under COVID-19: Social role disruptions, self-authenticity and present-focused coping
Source: PLoS One. 2021 Sep 3;16(9):e0256939. doi: 10.1371/journal.pone.0256939 (PMC8415594; doi:10.1371/journal.pone.0256939)
Supplement: S1 File — (DOCX) [file pone.0256939.s001.docx]

# S1 File

## S1 Table: Demographic characteristics of participants

|  | *N* (usable responses) | Gender | Age |
| --- | --- | --- | --- |
| Study 1 | 212 American Mturkers | 38.1% females | *M* = 37.18, *S.D.* = 11.17 |
| Study 2 | 289 American Mturkers | 37.4% females | *M* = 37.39, *S.D.* = 10.37 |
| Study 3 | 435 American Mturkers | 47.8% females | *M* = 37.97, *S.D.* = 11.57 |
| Study 4 | 291 HK university staffs and students | 65.3% females | *M* = 25.84, *S.D.* = 9.69 |

## S2 Table: Descriptive summary of role changes

| Study 1  (N = 212 American Mturkers) | *n*  (role applicable) | Role Change: 1 = my role had no change, 7 = my role had significant change | | |
| --- | --- | --- | --- | --- |
|  |  | *Mean* | | *S.D.* |
| Overall Role Change Index (average of all roles) | **212** | **4.16** | | **1.90** |
| Parent | 155 | 4.78 | | 2.20 |
| Child | 141 | 4.32 | | 2.15 |
| Employee | 189 | 4.71 | | 2.05 |
| Employer/supervisor | 149 | 4.66 | | 1.96 |
| Student | 118 | 4.77 | | 1.99 |
| Spouse/partner | 170 | 4.27 | | 2.26 |
| Friend | 196 | 4.13 | | 2.17 |
| Sibling | 188 | 3.94 | | 2.21 |
| Other: ______ | 115 | 3.91 | | 2.49 |
| Study 2  (N = 289 American Mturkers) | *Most Changed Role*  *%* | | *Least Changed Role*  *%* | |
| Parent/grandparent | 25.3 | | 15.6 | |
| Child/grandchild | 6.9 | | 7.3 | |
| Employee | 40.1 | | 24.6 | |
| Employer/supervisor | 11.1 | | 11.8 | |
| Student | 1.0 | | 3.1 | |
| Romantic partner (spouse/boyfriend/girlfriend) | 5.9 | | 20.8 | |
| Friend | 5.5 | | 11.1 | |
| Sibling | 1.7 | | 5.2 | |
| Other: ______ | 2.4 | | .7 | |
| Across studies, examples of “other” roles include uncle/aunt, neighbor, pet owner, sports fan, freelancer, customer, volunteer, citizen, etc. | | | | |
| Study 3  (N = 435 American Mturkers) | *n*  (role applicable) | Role Change: 1 = my role had no change, 7 = my role had significant change | | |
|  |  | *Mean* | | *S.D.* |
| Overall Role Change Index (average of all roles) | **435** | **3.83** | | **1.81** |
| Parent/grandparent | 278 | 4.28 | | 2.32 |
| Child/grandchild | 333 | 3.36 | | 2.23 |
| Employee | 382 | 4.57 | | 2.21 |
| Employer/supervisor | 238 | 4.62 | | 2.16 |
| Student | 186 | 4.57 | | 2.21 |
| Romantic partner (spouse/boyfriend/girlfriend) | 369 | 3.78 | | 2.25 |
| Friend | 419 | 3.88 | | 2.14 |
| Sibling | 388 | 3.39 | | 2.25 |
| Other: ______ | 159 | 4.21 | | 2.51 |
| Study 4  (N = 291 Hong Kong university staffs & students) | *n*  (role applicable) | Role Change: 1 = my role had no change, 7 = my role had significant change | | |
|  |  | *Mean* | | *S.D.* |
| Overall Role Change Index (average of all roles) | **291** | **3.48** | | **1.50** |
| Parent/grandparent | 73 | 3.07 | | 2.12 |
| Child/grandchild | 268 | 2.86 | | 1.89 |
| Employee | 191 | 3.92 | | 2.14 |
| Employer/supervisor | 71 | 3.46 | | 2.16 |
| Student | 170 | 3.34 | | 2.00 |
| Romantic partner (spouse/boyfriend/girlfriend) | 289 | 3.66 | | 1.89 |
| Friend | 226 | 2.83 | | 1.94 |
| Sibling | 217 | 4.39 | | 1.89 |
| Other: ______ | 67 | 3.51 | | 2.18 |
|  |  |  | |  |

## S3 Table: Correlations between change in each role and inauthenticity in Study 1

|  | Correlation with inauthenticity (Pearson’s r) | *N* selected the role | 95% CI | *p*-value |
| --- | --- | --- | --- | --- |
| *Study 1 (MTurk American residents)* | | | | |
| Role Change Index | **.59** | **212** | **[.493, .671]** | **<.001** |
| parent | .57 | 155 | [.455, .669] | <.001 |
| child | .59 | 141 | [.474, .690] | <.001 |
| employee | .34 | 189 | [.203, .457] | <.001 |
| employer/supervisor | .47 | 149 | [.337, .588] | <.001 |
| spouse/partner | .66 | 170 | [.560, .733] | <.001 |
| friend | .57 | 196 | [.465, .656] | <.001 |
| sibling | .61 | 188 | [.507, .689] | <.001 |
| student | .59 | 118 | [.462, .699] | <.001 |
| other | .48 | 115 | [.328, .611] | <.001 |

## S4 Table: Regression analyses with additional covariates in Study 1

|  |  | **DV = Inauthenticity** |
| --- | --- | --- |
| ***IV*** | Adjusted *R^2^* | .67 |
| **Role Change** | Standardized *β* | .35 |
|  | *t*-statistics | 7.42 |
|  | *p*-value | <.001 |
|  | 95% CI | [.521, .898] |
|  | *VIF* | 1.31 |
| **Self-esteem** | Standardized *β* | -.56 |
|  | *t*-statistics | -11.67 |
|  | *p*-value | <.001 |
|  | 95% CI | [-1.326, -.943] |
|  | *VIF* | 1.35 |
| **Gender (male=0, female=1)** | Standardized *β* | .02 |
|  | *t*-statistics | .54 |
|  | *p*-value | .59 |
|  | 95% CI | [-.264, .462] |
|  | *VIF* | 1.15 |
| **Income** | Standardized *β* | -.02 |
|  | *t*-statistics | -.55 |
|  | *p*-value | .59 |
|  | 95% CI | [-.222, .126] |
|  | *VIF* | 1.10 |
| **Age** | Standardized *β* | .03 |
|  | *t*-statistics | .66 |
|  | *p*-value | .51 |
|  | 95% CI | [-.118, .236] |
|  | *VIF* | 1.15 |
| **Knows Someone with COVID (yes=1, no=0)** | Standardized *β* | .08 |
|  | *t*-statistics | 1.69 |
|  | *p*-value | .09 |
|  | 95% CI | [-.064, .830] |
|  | *VIF* | 1.40 |
| **Has Chronic Diseases (yes=1, no=0)** | Standardized *β* | -.04 |
|  | *t*-statistics | -.92 |
|  | *p*-value | .36 |
|  | 95% CI | [-.659, .240] |
|  | *VIF* | 1.27 |
| **Essential worker (yes = 1, no = 0)** | Standardized *β* | .08 |
|  | *t*-statistics | 1.77 |
|  | *p*-value | .08 |
|  | 95% CI | [-.040, .742] |
|  | *VIF* | 1.17 |

## S5 Table: Regression analyses of self-esteem and mood (as DVs and as covariates), Study 2

|  |  | **DV = self-esteem** | **DV = mood** | **DV = inauthenticity** | **DV = inauthenticity** |
| --- | --- | --- | --- | --- | --- |
| ***IV*** | Adjusted *R^2^* | .07 | .23 | .52 | .17 |
| **Role condition (1 = changed, -1 = constant)** | Standardized *β* | .03 | .10 | .06 | .03 |
|  | *t*-statistics | .46 | 1.82 | 1.34 | .58 |
|  | *p*-value | .65 | .07 | .18 | .56 |
|  | 95% CI | [-.113, .182] | [-.010, .242] | [-.054, .285] | [-.159, .292] |
|  | *VIF* | 1.05 | 1.05 | 1.05 | 1.06 |
| **Role centrality** | Standardized *β* | .27 | -.01 | -.09 | -.26 |
|  | *t*-statistics | 4.68 | -.17 | -2.17 | -4.76 |
|  | *p*-value | .00 | .87 | .03 | .00 |
|  | 95% CI | [.202, .494] | [-.135, .114] | [-.367, -.018] | [-.760, -.316] |
|  | *VIF* | 1.03 | 1.03 | 1.11 | 1.03 |
| **Role condition X Role centrality** | Standardized *β* | -.06 | .04 | .08 | .12 |
|  | *t*-statistics | -1.05 | .70 | 2.00 | 2.15 |
|  | *p*-value | .29 | .48 | .05 | .03 |
|  | 95% CI | [-.224, .068] | [-.080, .169] | [.002, .339] | [.021, .465] |
|  | *VIF* | 1.02 | 1.02 | 1.02 | 1.02 |
| **Valence of COVID-19’s impact** | Standardized *β* | -.12 | .51 | .28 | .32 |
|  | *t*-statistics | -2.12 | 9.52 | 6.63 | 5.03 |
|  | *p*-value | .03 | < .001 | < .001 | < .001 |
|  | 95% CI | [-.309, -.012] | [.485, .738] | [.408, .752] | [.403, .921] |
|  | *VIF* | 1.06 | 1.06 | 1.07 | 1.40 |
| **Self-esteem** | Standardized *β* |  |  | -.62 |  |
|  | *t*-statistics |  |  | -14.62 |  |
|  | *p*-value |  |  | .00 |  |
|  | 95% CI |  |  | [-1.461, -1.114] |  |
|  | *VIF* |  |  | 1.09 |  |
| **Mood** | Standardized *β* |  |  |  | .07 |
|  | *t*-statistics |  |  |  | 1.20 |
|  | *p*-value |  |  |  | .23 |
|  | 95% CI |  |  |  | [-.098, .407] |
|  | *VIF* |  |  |  | 1.32 |

## S6 Table: Regression analyses of self-esteem and mood (as DVs and as covariates), Study 3

|  |  | **DV = self-esteem** | **DV = mood** | **DV = inauthenticity** | **DV = inauthenticity** | **DV = inauthenticity** |
| --- | --- | --- | --- | --- | --- | --- |
| ***IV*** | Adjusted *R^2^* | .14 | .18 | .36 | .68 | .40 |
| **Role change** | Standardized *β* | -.23 | .04 | .44 | .24 | .38 |
|  | *t*-statistics | -2.72 | .54 | 6.20 | 4.69 | 5.45 |
|  | *p*-value | .01 | .59 | .00 | .00 | .00 |
|  | 95% CI | [-.507, -.081] | [-.156, .273] | [.589, 1.136] | [.273, .668] | [.447, 1.015] |
|  | *VIF* | 3.57 | 3.57 | 3.43 | 3.63 | 3.57 |
| **Past focus** | Standardized *β* | -.07 | .02 | .08 | .04 | .09 |
|  | *t*-statistics | -1.37 | .41 | 1.78 | 1.41 | 2.06 |
|  | *p*-value | .17 | .68 | .08 | .16 | .04 |
|  | 95% CI | [-.474, .085] | [-.223, .339] | [-.034, .697] | [-.073, .441] | [.018, .723] |
|  | *VIF* | 1.39 | 1.39 | 1.39 | 1.39 | 1.39 |
| **Future focus** | Standardized *β* | -.01 | -.03 | -.02 | -.02 | -.02 |
|  | *t*-statistics | -.12 | -.63 | -.47 | -.49 | -.38 |
|  | *p*-value | .90 | .53 | .64 | .63 | .71 |
|  | 95% CI | [-.296, .261] | [-.371, .191] | [.452, .278] | [-.320, .193] | [-.420, .285] |
|  | *VIF* | 1.39 | 1.39 | 1.38 | 1.39 | 1.39 |
| **Role change X Past focus** | Standardized *β* | -.13 | -.01 | .12 | .07 | .14 |
|  | *t*-statistics | -1.94 | -.10 | 2.13 | 1.64 | 2.55 |
|  | *p*-value | .05 | .92 | .03 | .10 | .01 |
|  | 95% CI | [-.565, .004] | [-.302, .271] | [.031, .775] | [-.043, .482] | [.107, .826] |
|  | *VIF* | 2.20 | 2.20 | 2.18 | 2.22 | 2.20 |
| **Role change X Future focus** | Standardized *β* | -.11 | .03 | .13 | .08 | .15 |
|  | *t*-statistics | -1.71 | .44 | 2.21 | 2.01 | 2.75 |
|  | *p*-value | .09 | .66 | .03 | .04 | .01 |
|  | 95% CI | [-.527, .036] | [-.221, .346] | [.046, .782] | [.006, .525] | [.142, .854] |
|  | *VIF* | 2.25 | 2.25 | 2.24 | 2.27 | 2.25 |
| **Valence of role change** | Standardized *β* | .01 | .42 |  | .18 | .24 |
|  | *t*-statistics | .11 | 9.38 |  | 6.56 | 5.75 |
|  | *p*-value | .91 | < .001 |  | < .001 | < .001 |
|  | 95% CI | [-.110, .123] | [.442, .677] |  | [.250, .464] | [.311, .634] |
|  | *VIF* | 1.07 | 1.07 |  | 1.07 | 1.29 |
| **Self-esteem** | Standardized *β* |  |  |  | -.59 |  |
|  | *t*-statistics |  |  |  | -2.12 |  |
|  | *p*-value |  |  |  | .00 |  |
|  | 95% CI |  |  |  | [-1.262, -1.037] |  |
|  | *VIF* |  |  |  | 1.18 |  |
| **Mood** | Standardized *β* |  |  |  |  | -.15 |
|  | *t*-statistics |  |  |  |  | -3.59 |
|  | *p*-value |  |  |  |  | < .001 |
|  | 95% CI |  |  |  |  | [-.448, -.131] |
|  | *VIF* |  |  |  |  | 1.24 |

## S7 Table: Valence of role change as an additional moderator in Study 3

| **DV = inauthenticity** | *Standardized β* | *t-statistics* | *p-value* | *95% CI* | *VIF* |
| --- | --- | --- | --- | --- | --- |
| Role change | .340 | 4.657 | < .001 | [.383,.942] | 3.92 |
| Valence of role change | .125 | 3.101 | .002 | [.089,.397] | 1.19 |
| Past focus | .105 | 2.343 | .02 | [.070,.795] | 1.49 |
| Future focus | -.010 | -.217 | .83 | [-.402,.322] | 1.49 |
| Role change X Past focus | .168 | 2.991 | .003 | [.191,.925] | 2.32 |
| Role change X Future focus | .147 | 2.566 | .01 | [.112,.845] | 2.43 |
| Role change X Valence of role change | .177 | 2.424 | .02 | [.063,.603] | 3.93 |
| Role change X Past focus X Valence of role change | -.061 | -1.090 | .28 | [-.555,.159] | 2.33 |
| Role change X Future focus X Valence of role change | .047 | .797 | .43 | [-.209,.494] | 2.51 |

## S8 Table: Regression analyses with additional covariates in Study 4

|  |  | **DV = Inauthenticity** |
| --- | --- | --- |
| **IV** | Adjusted *R^2^* | .47 |
| **Role change** | Standardized *β* | .18 |
|  | *t*-statistics | 3.73 |
|  | *p*-value | < .001 |
|  | 95% CI | [.104, .337] |
|  | *VIF* | 1.18 |
| **Present focus** | Standardized *β* | -.08 |
|  | *t*-statistics | -1.70 |
|  | *p*-value | .09 |
|  | 95% CI | [-.208, .016] |
|  | *VIF* | 1.09 |
| **Role change X Present focus** | Standardized *β* | -.09 |
|  | *t*-statistics | -1.92 |
|  | *p*-value | .06 |
|  | 95% CI | [-.211, .003] |
|  | *VIF* | 1.04 |
| **Self-esteem** | Standardized *β* | -.44 |
|  | *t*-statistics | -8.57 |
|  | *p*-value | < .001 |
|  | 95% CI | [-.651, -.408] |
|  | *VIF* | 1.30 |
| **Valence of role change** | Standardized *β* | -.04 |
|  | *t*-statistics | -.86 |
|  | *p*-value | .39 |
|  | 95% CI | [-.160, .062] |
|  | *VIF* | 1.16 |
| **Pre-Covid inauthenticity** | Standardized *β* | .31 |
|  | *t*-statistics | 6.19 |
|  | *p*-value | < .001 |
|  | 95% CI | [.251, .486] |
|  | *VIF* | 1.24 |
| **Physical appearance change** | Standardized *β* | .04 |
|  | *t*-statistics | .85 |
|  | *p*-value | .40 |
|  | 95% CI | [-.069, .174] |
|  | *VIF* | 1.15 |
| **Valence of physical change** | Standardized *β* | .03 |
|  | *t*-statistics | .68 |
|  | *p*-value | .50 |
|  | 95% CI | [-.074, .151] |
|  | *VIF* | 1.14 |
| **Gender (1 = male, 0= female)** | Standardized *β* | -.04 |
|  | *t*-statistics | -.91 |
|  | *p*-value | .36 |
|  | 95% CI | [-.331, .121] |
|  | *VIF* | 1.05 |
| **Age** | Standardized *β* | -.09 |
|  | *t*-statistics | -1.84 |
|  | *p*-value | .07 |
|  | 95% CI | [-.025, .001] |
|  | *VIF* | 1.18 |

## S9 Table: Correlations between change in each role and inauthenticity in Study 4

| Specific Role Change | Correlation with inauthenticity (Pearson’s r) | *N* selected the role | 95% CI | *p*-value |
| --- | --- | --- | --- | --- |
| *Study 3 (Hong Kong university staff and students)* | | | | |
| Role Change Index | .214 | 291 | [.102, .321] | < .001 |
| parent/grandparent | -.045 | 73 | [-.272, .187] | .703 |
| child/grandchild | .162 | 268 | [.043, .276] | .008 |
| employee | .137 | 191 | [-.005, .274] | .058 |
| employer/supervisor | .049 | 71 | [-.186, .279] | .683 |
| student | .239 | 170 | [.092, .376] | .002 |
| spouse/partner/boyfriend/girlfriend | .172 | 289 | [.058, .282] | .003 |
| friend | .167 | 226 | [.037, .291] | .012 |
| sibling | .205 | 217 | [.074, .329] | .002 |
| other | -.054 | 67 | [-.290, .187] | .664 |
| *Note*: We suspect that the correlation for the employee role was weaker in study 3, in part, because the majority of participants were students and, hence, the role as an employee was less central to their sense of self. As the result of study 2 would suggest, this low level of centrality would weaken the effect of role change on authenticity. We suspect that the correlations for employer/supervisor and for parent/grandparent were weaker in study 3 because the sample sizes of those who selected these roles were small. | | | | |

## S10 Table: Analyses with present-, past- and future-focus as moderators in Study 4

| **DV = inauthenticity** | | “Temporal focus” = present | “Temporal focus” = past | “Temporal focus” = future |
| --- | --- | --- | --- | --- |
| ***IV*** | Adjusted *R^2^* | .46 | .44 | .45 |
| **Role change** | Standardized *β* | .190 | .163 | .179 |
|  | *t*-statistics | 4.193 | 3.558 | 3.883 |
|  | *p*-value | < .001 | < .001 | < .001 |
|  | 95% CI | [.121, .335] | [.087, .303] | [.106, .323] |
|  | *VIF* | 1.098 | 1.092 | 1.113 |
| **Temporal focus** | Standardized *β* | -.095 | .022 | -.038 |
|  | *t*-statistics | -2.135 | .487 | -.841 |
|  | *p*-value | .034 | .627 | .401 |
|  | 95% CI | [-.218, -.009] | [-.081, .134] | [-.152, .061] |
|  | *VIF* | 1.051 | 1.080 | 1.064 |
| **Role change X Temporal focus** | Standardized *β* | -.075 | .019 | -.055 |
|  | *t*-statistics | -1.711 | .432 | -1.257 |
|  | *p*-value | .088 | .666 | .210 |
|  | 95% CI | [-.185, .013] | [-.078, .122] | [-.159, .035] |
|  | *VIF* | 1.011 | 1.016 | 1.015 |
| **Self-esteem** | Standardized *β* | -.432 | -.432 | -.433 |
|  | *t*-statistics | -9.062 | -8.946 | -8.983 |
|  | *p*-value | < .001 | < .001 | < .001 |
|  | 95% CI | [-.629, -.405] | [-.630, -.403] | [-.631, -.404] |
|  | *VIF* | 1.211 | 1.212 | 1.215 |
| **Valence of role change** | Standardized *β* | -.040 | -.036 | -.043 |
|  | *t*-statistics | -.899 | -.796 | -.957 |
|  | *p*-value | .370 | .427 | .339 |
|  | 95% CI | [-.152, .057] | [-.150, .064] | [-.156, .054] |
|  | *VIF* | 1.047 | 1.072 | 1.042 |
| **Pre-Covid inauthenticity** | Standardized *β* | .322 | .325 | .320 |
|  | *t*-statistics | 6.720 | 6.699 | 6.621 |
|  | *p*-value | < .001 | < .001 | < .001 |
|  | 95% CI | [.272, .498] | [.275, .504] | [.269, .497] |
|  | *VIF* | 1.223 | 1.226 | 1.224 |

## S1 Appendix: Normal P-P plot of regression standardized residual and scatterplot of residuals

### Study 1: Equation 1


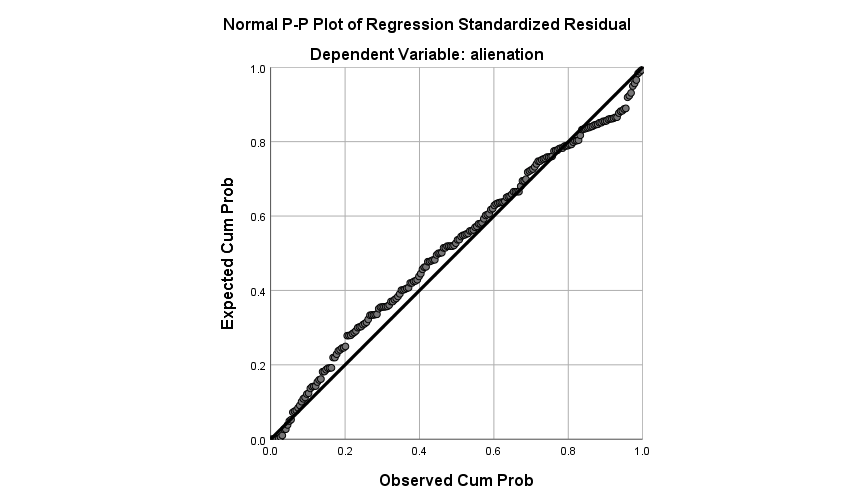


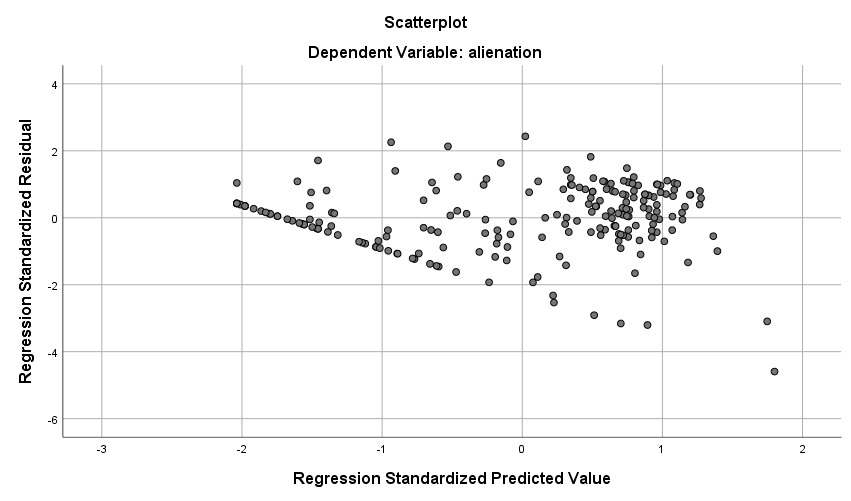


### Study 2: Equation 2


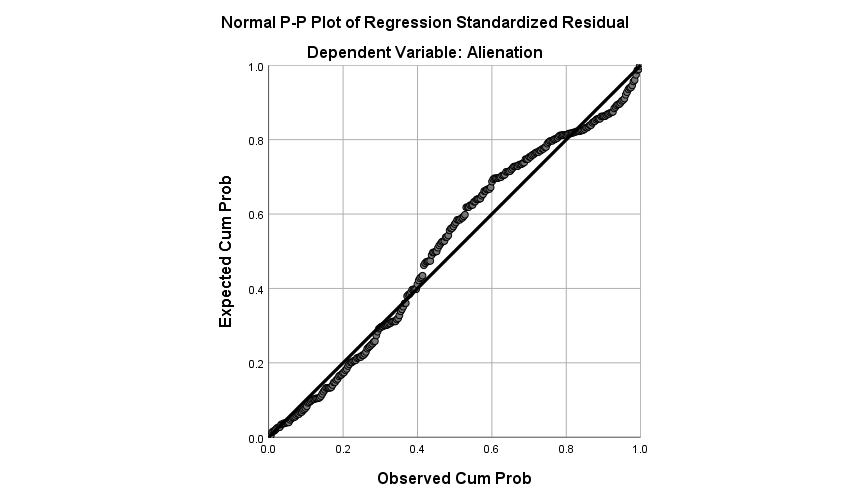

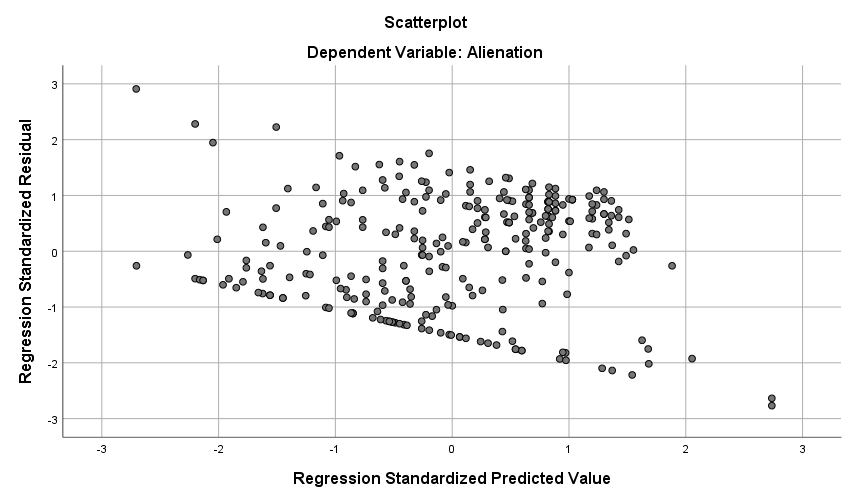


### Study 3: Equation 3


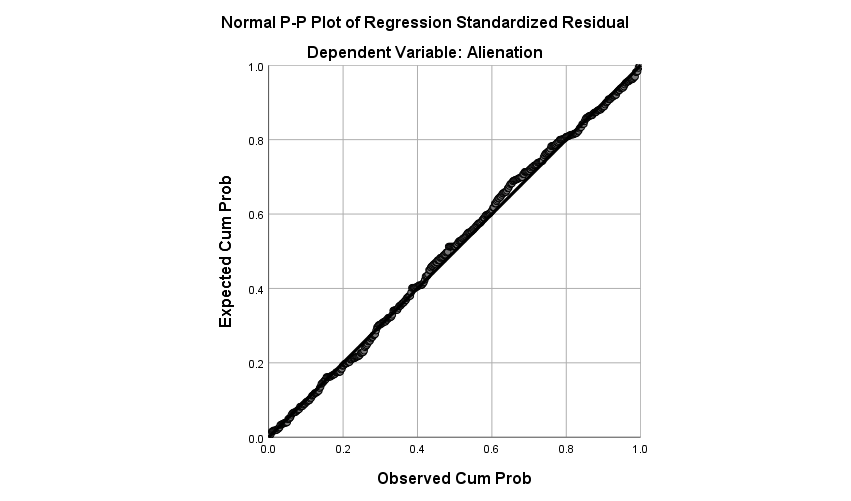

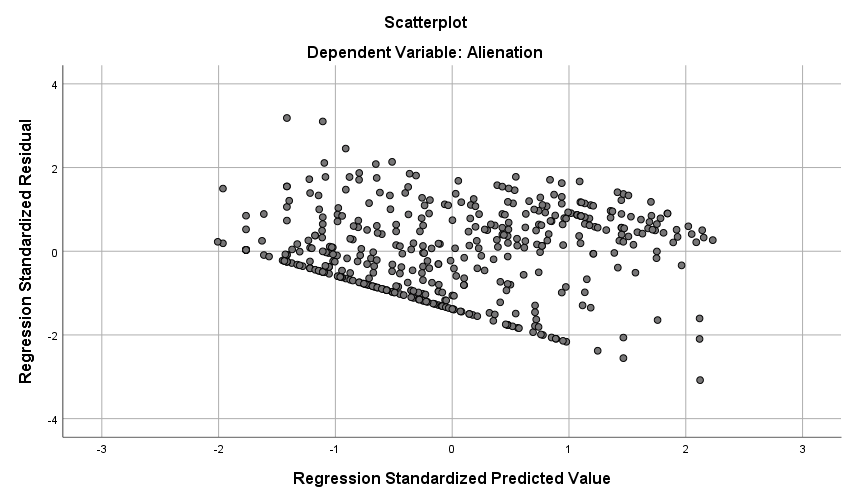


### Study 4:

***Equation 4 (main effect)***


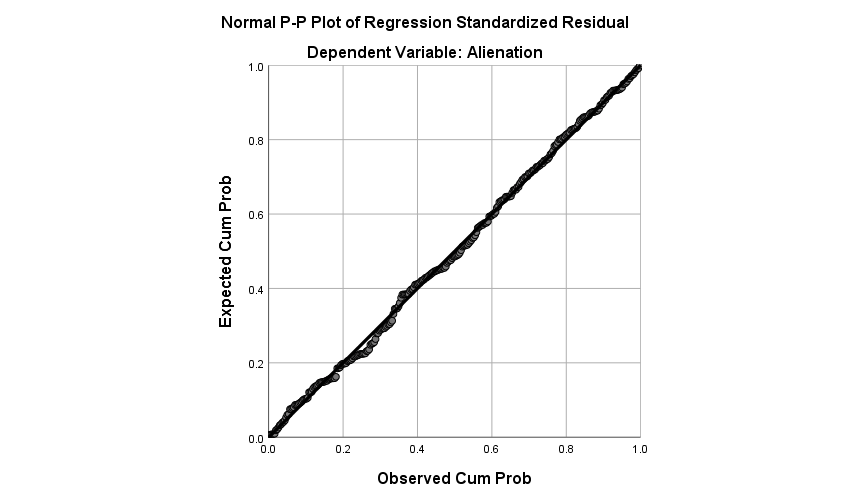

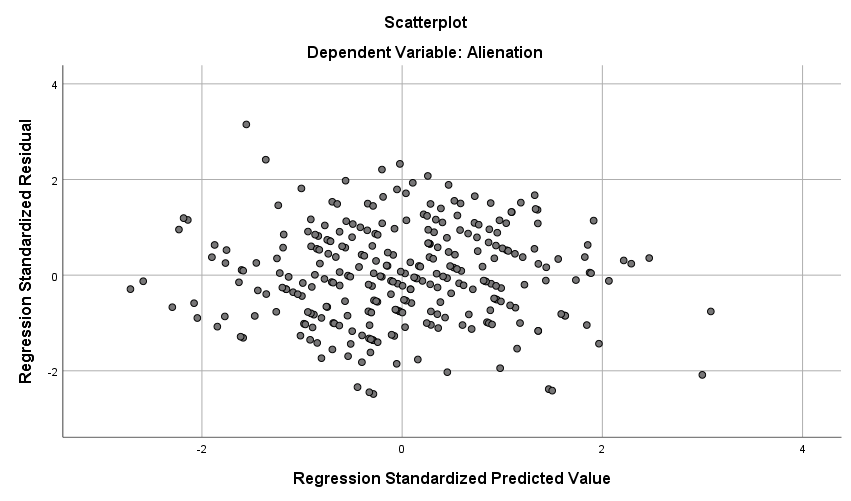


***Equation 5 (interaction with present focus)***


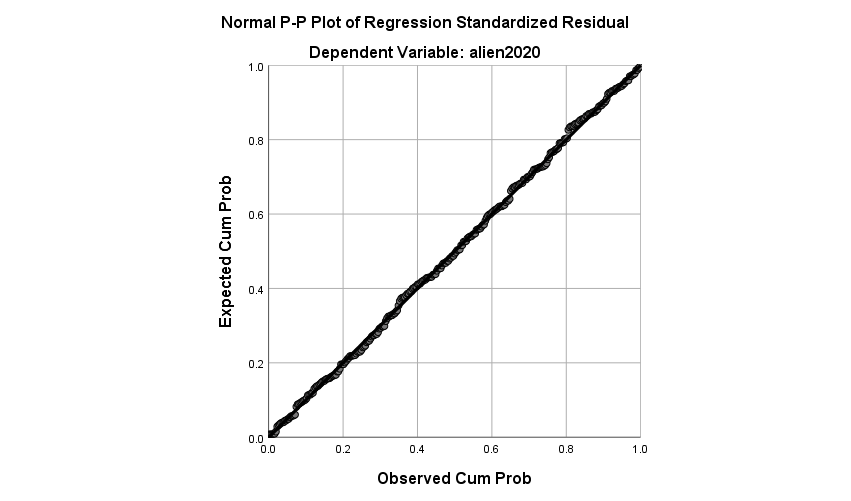

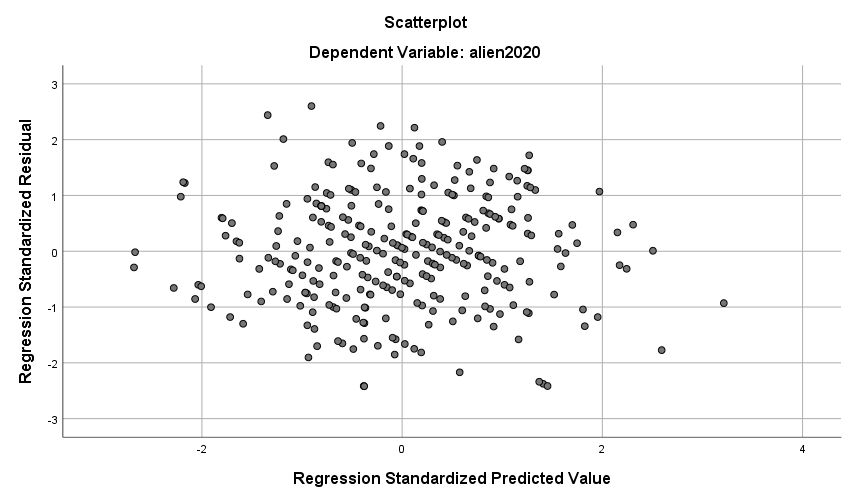


## S2 Appendix: Stimuli in studies

### 1. Role change measure (Studies 1, 3, 4)

Since the COVID-19 outbreak began, some people’s roles at home or at work have changed significantly (e.g., a parent may be caring for children while working from home; friends may be socializing remotely and online; a worker may be left with no work). Other people’s roles at home or at work have changed less.

Please think about the different roles you play in your life and the tasks you perform in each of those roles. How much change are you experiencing in each role?

(Please select N/A for roles you do not have).

- As a parent/grandparent
- As a child/grandchild
- As an employee
- As an employer/supervisor
- As a student
- As a romantic partner (spouse/boyfriend/girlfriend)
- As a friend
- As a sibling
- Other, as a(n) ________ (e.g., teammate, club member, coach, etc...). If all your roles are listed above, please select N/A)

1 = My role has no change, 7 = My role has significant changes

### 2. Authenticity Scale (full scale)

(Items 1-4 are the self-alienation subscale that measures feelings of inauthenticity in our studies).

Please indicate how much you agree with each of the following statements.

1. I feel out of touch with the “real me.”
2. I feel as if I don’t know myself very well.
3. I feel alienated from myself.
4. I don’t know how I really feel inside.
5. I always stand by what I believe in.
6. I am true to myself in most situations.
7. I think it is better to be yourself, than to be popular.
8. I live in accordance with my values and beliefs.
9. I usually do what other people tell me to do.
10. Other people influence me greatly.
11. I am strongly influenced by the opinions of others.
12. I always feel I need to do what others expect me to do.

1 = Not at all, 7 = Very much / 1 = Strongly agree, 7 = Strongly disagree

### 3. Attention check embedded in self-authenticity scale (studies 1, 2, 3)

- This is an attention check, please select the option that indicates “very much”/ “strongly agree”.

### 4. Self-esteem scale [1]

How much do you agree or disagree with the following statements?

- On the whole, I am satisfied with myself.
- At times I think I am no good at all.
- I feel that I have a number of good qualities.
- I am able to do things as well as most other people
- I feel I do not have much to be proud of.
- I certainly feel useless at times.
- I feel that I’m a person of worth.
- I wish I could have more respect for myself.
- All in all, I am inclined to think that I am a failure.
- I take a positive attitude toward myself.

1 = Strongly disagree, 4 = Strongly agree

### 5. Role change manipulation (Study 2)

*(changed condition)*Now, please describe how your role as [most changed role] has changed, since the COVID-19 outbreak. For example, please describe the change in your responsibilities, your surrounding environment, the amount of time you spend and when you spend that time, or any other relevant aspects of this role that have changed for you.

Please provide detailed descriptions. This task should take you a few minutes to complete but you may spend as long as you like to complete it.

*(constant condition)*Now, please describe how your role as [least changed role] has remained the same, since the COVID-19 outbreak. For example, please describe your responsibilities, your surrounding environment, the amount of time you spend and when you spend that time, or any other relevant aspects of this role that remain the same for you.

Please provide detailed descriptions. This task should take you a few minutes to complete but you may spend as long as you like to complete it.

### 6. Strength of Identification scale (adapted from [2]; Study 2)

Please indicate to what extent you agree or disagree with the statements below.

***Centrality (α = .66)***

- I often think about being [role].
- Being [role] has little to do with how I feel about myself in general.
- Being [role] is an important part of my self image.
- The fact that I am [role] rarely enters my mind.

Other items in the Strength of Identification scale (whole scale *α* = .79)

*Affect*

- In general I am glad to be [role].
- I often regret being [role].
- Generally I feel good about myself when I think about being [role].
- I do not feel good about being [role].

*Tie*

- I have a lot in common with others who are also [role].
- I feel strong ties to others who are also [role].
- I find it difficult to form a bond with others who are also [role].
- I do not feel a strong sense of being connected to others who are also [role].

1 = Strongly disagree, 7 = Strongly agree

### 7. Valence of role change measure (Studies 3-4)

Overall, how do you feel about the changes in your roles due to COVID-19?
1= Very negative, 7 = Very positive

### 8. Temporal focus manipulation (Study 3)

*(past condition)*In the text-box below, please describe how COVID-19 has made you focus on the PAST (i.e., life before COVID-19). Please provide as much detail as you can.

*(present condition)*In the text-box below, please describe how COVID-19 has made you focus on the PRESENT (i.e., one day at a time during COVID-19). Please provide as much detail as you can.

*(future condition)*In the text-box below, please describe how COVID-19 has made you focus on the FUTURE (i.e., life after COVID-19). Please provide as much detail as you can.

### 9. Temporal focus measure (Study 4)

Since the COVID-19 outbreak, how often do you think about…

- Life in the PAST, before COVID-19
- Life in the PRESENT, during COVID-19
- Life in the FUTURE, after COVID-19

1 = Never, 5 = All the time

## S3 Appendix: Analyses using the full inauthenticity scale

We obtained consistent results whether using the full scale or the self-alienation subscale as the DV. There were three exceptions: study 2’s simple effect of role change at +1 *SD* of role centrality (which was weakened when the full scale was the DV), study 3’s interaction of role change and present-focus (which was weakened when the full scale was the DV), and study 4’s simple contrast between present-focus and future-focus at +1 *SD* of role change (which was strengthened when the full scale was the DV). We elaborated on all results below.

### Study 1

We find that role change correlated positively with inauthenticity (*r* = .57, *p* < .001, 95% CI = [.471, .654]). We also separately computed correlations for each role (see Table below). Because self-esteem often correlates negatively with inauthenticity [3], we also regressed inauthenticity on role change, keeping self-esteem as a covariate in the correlational studies (i.e., here and Study 3; scale measures were standardized in analyses in all studies). Role change positively predicted inauthenticity (*β* = .32, *t*(209) = 7.30, *p* < .001, 95% CI = [.308, .536]), even controlling for self-esteem, which negatively predicted inauthenticity (*β* = -.64, *t*(209) = -14.58, *p* < .001, 95% CI = [-.957, -.729]), attesting to the discriminant construct validity between inauthenticity and self-esteem.

| Specific Role Change | Correlation with inauthenticity (Pearson’s r) | Number of participants selected this role (n) | 95% CI | *p*-value |
| --- | --- | --- | --- | --- |
| ***Study 1 (MTurk American residents)*** | | | |  |
| parent | .557 | 155 | [.438, .657] | <.001 |
| child | .592 | 141 | [.473, .690] | <.001 |
| employee | .330 | 189 | [.197, .451] | <.001 |
| employer/supervisor | .427 | 149 | [.286, .550] | <.001 |
| spouse/partner | .629 | 170 | [.529, .712] | <.001 |
| friend | .561 | 196 | [.457, .650] | <.001 |
| sibling | .590 | 188 | [.488, .676] | <.001 |
| student | .602 | 118 | [.473, .706] | <.001 |
| other | .480 | 115 | [.325, .610] | <.001 |
| ***Study 4 (Hong Kong university staff and students)*** | | | |  |
| parent/grandparent | -.072 | 73 | [-.297, .161] | .546 |
| child/grandchild | .157 | 268 | [.038, .272] | .010 |
| employee | .170 | 191 | [.029, .305] | .019 |
| employer | -.007 | 71 | [-.240, .227] | .955 |
| student | .203 | 170 | [.054, .343] | .008 |
| partner | .162 | 289 | [.048, .272] | .006 |
| friend | .144 | 226 | [.014, .269] | .031 |
| sibling | .215 | 217 | [.084, .338] | .001 |
| other | .024 | 67 | [-.217, .263] | .846 |

### Study 2

We regressed inauthenticity on role (1 = changed, -1 = constant), role centrality, and their interaction, with valence of impact as a covariate. Results revealed a predicted interaction (*β* = .12, *t*(284) = 2.14, *p* = .03, 95% CI = [.012, .288]), qualifying main effects of role centrality (*β* = -.23, *t*(284) = -4.13, *p* < .001, 95% CI = [-.429, -.152]) and valence of impact (*β* = .32, *t*(284) = 5.59, *p* < .001, 95% CI = [.258, .539]). Consistent with our theorizing, spotlight analysis showed that role change (vs. constancy) increased inauthenticity under when a role is highly central to the self (+1 *S.D.*, *b* = .17, *SE* = .10, *t*(284) = 1.68, *p* = .094, 95% CI = [-.029, .364]); but not when the role is not central to the self (-1 *S.D.*, *b* = -.13, *SE* = .10, *t*(284) = -1.33, *p* = .185, 95% CI = [-.328, .064]).

### Study 3

Treating present-focus as the benchmark, we regressed inauthenticity on role change, past-focus (1 = yes, 0 = otherwise), future-focus (1 = yes, 0 = otherwise), role change by past-focus interaction, and role change by future-focus interaction, with valence of role change as a covariate (as in Study 3). Results revealed both role change by past-focus (*β* = .17, *t*(428) = 2.83, *p* = .005, 95% CI = [.109, .605]) and role change by future-focus (*β* = .16, *t*(428) = 2.63, *p* = .009, 95% CI = [.083, .574]) interactions, qualifying main effects of role change (*β* = .33, *t*(428) = 4.42, *p* < .001, 95% CI = [.232, .603]) and valence of role change (*β* = .12, *t*(428) = 3.02, *p* = .003, 95% CI = [.055, .258]).

Slope analysis revealed that role change increased inauthenticity in all three conditions, but least in the present-focus condition (present: *b* = .42, *SE* = .09, *t*(428) = 4.42, *p* < .001, 95% CI = [.232, .603]; past: *b* = .77, *SE* = .09, *t*(428) = 9.05, *p* < .001, 95% CI = [.607, .943]; future: *b* = .75, *SE* = .08, *t*(428) = 8.92, *p* < .001, 95% CI = [.582, .911]), and the interactions indicated that the slopes differed. In addition, spotlight analysis revealed that under high role change (+1 *S.D*.), past-focus (*b* = .53, *SE* = .17, *t*(428) = 3.04, *p* = .003, 95% CI = [.187, .873]) and, directionally, future-focus (*b* = .19, *SE* = .17, *t*(428) = 1.08, *p* = .28, 95% CI = [-.153, .527]) conditions felt more inauthentic than the present-focus condition. Under low role change (-1 *S.D*), however, future-focus reduced inauthenticity (*b* = -.47, *SE* = .18, *t*(428) = -2.64, *p* = .009, 95% CI = [-.821, -.120]; past vs. present: *b* = -.18, *SE* = .18, *t*(428) = -1.03, *p* = .30).

### Study 4

***Main Effect*.** Role change and inauthenticity were positively correlated (*r* = .21, *p* < .001, 95% CI = [.095, .315]), replicating Study 1 and showing generalizability of the effect to a different population. In addition, we regressed inauthenticity on role change, with self-esteem, valence of change, and pre-COVID-19 inauthenticity as covariates. As predicted, the effect of role change remained (*β* = .14, *t*(286) = 3.28, *p* = .001, 95% CI = [.047, .187]). Thus, COVID-19-related role changes heighten inauthenticity independent of self-esteem (*β* = -.38, *t*(286) = -8.30, *p* = .001, 95% CI = [-.400, -.247]), valence of the role change (*β* = -.03, *t*(286) = -.62, *p* = .536, 95% CI = [-.093, .048]), and pre-COVID-19 inauthenticity (*β* = .44, *t*(286) = 9.51, *p* = .001, 95% CI = [.297, .452]).

***Present-Focused Coping*.** We regressed inauthenticity on role change, present-focus, and their interaction, with above covariates. Results yielded an interaction of role change and present-focus (*β* = -.09, *t*(284) = -2.25, *p* = .03, 95% CI = [-.142, -.009]), qualifying main effects of role change (*β* = .16, *t*(284) = 3.77, *p* < .001, 95% CI = [.065, .207]), present-focus (*β* = -.09, *t*(284) = -2.17, *p* = .03, 95% CI = [-.145, -.006]), self-esteem (*β* = -.38, *t*(284) = -8.32, *p* < .001, 95% CI = [-.397, -.245]), and pre-COVID-19 inauthenticity (*b* = .45, *t*(284) = 9.76, *p* < .001, 95% CI = [.304, .457]).

Simple effect analysis showed that role change predicted inauthenticity (*b* = .21, *SE* = .05, *t*(284) = 4.21, *p* < .001, 95% CI = [.113, .311]) among those who tended not to focus on the present (-1 *S.D.*). This effect was offset for those who tended to focus on the present (+1 *S.D*.; *b* = .06, *SE* = .05, *t*(284) = 1.25, *p* = .214, 95% CI = [-.035, .156]). Moreover, a second simple effect analysis showed that present-focus reduced inauthenticity under high role change (+1 *S.D*.; *b* = -.15, *SE* = .05, *t*(284) = -3.12, *p* = .002, 95% CI = [-.246, -.056]), and not low role change (-1 *S.D*., *b* = .0003, *SE* = .05, *t*(284) = .006, *p* = .99, 95% CI = [-.096, .097]).

## S4 Appendix: Re-analyzing Study 2 using the Strength of Identification (SOI) full scale

We regressed inauthenticity on role (1 = changed, -1 = constant), SOI (standardized), and their interaction, with valence of COVID-19’s impact as a covariate. Results of the regression analysis revealed a predicted interaction (*β* = .15, *t*(284) = 3.40, *p* = .01, 95% CI = [.134, .502]), and main effects of SOI (*β* = -.57, *t*(284) = -12.70, *p* < .001, 95% CI = [-1.365, -.998]) and valence of impact (*β* =.34, *t*(284) = 7.36, *p* < .001, 95% CI = [.513, .887]). Simple effect analysis showed that role change (vs. constancy) increased inauthenticity when the roles were high in SOI (+1 *SD*: *b* = .41, *SE* = .13, *t*(284) = 3.09, *p* = .029, 95% CI = [.150, .678]), but not when the roles were low in SOI (-1 *SD*: *b* = -.22, *SE* = .13, *t*(284) = -1.68, *p* = .09, 95% CI = [-.481, .038]).

## S5 Appendix: Exploratory analyses

### Study 3

We measured individual differences (e.g., mindfulness, approach and avoidant coping, inclusion of others into the self, self-consciousness, etc.) as potential moderators and mediators of how COVID-related role changes may affect inauthenticity. We found a moderating effect of mindfulness and a mediating effect avoidant coping, which we detailed below. Because these variables were only measured in Study 3 and hence, unable to be tested and replicated in other studies, results should be interpreted with caution. All analyses are available upon request.

***Mindfulness*.** Mindfulness refers to an unbiased awareness of one’s present experience and situation [4]. A present-focus coping strategy might be more effective among those who are chronically mindful. Indeed, regression on inauthenticity with mindfulness as a second moderator yielded a 3-way interaction of past-focus X role change X mindfulness (*β* = .12, *t*(422) = 2.16, *p* = .03, 95% CI = [.033, .705]), 2-way interactions of past-focus X by role change (*β* = .13, *t*(422) = 2.54, *p* = .01, 95% CI = [.098, .770]), future-focus X role change (*β* = .15, *p* = .005, 95% CI = [.147, .828]), past-focus X mindfulness (*β* = .12, *t*(422) = 2.18, *p* = .030, 95% CI = [.037, .720]), and main effects of role change (*β* = .37, *t*(422) = 5.62, *p* < .001, 95% CI = [.471, .979]) and valence of role change (*β* = .22, *t*(422) = 6.12, *p* < .001, 95% CI = [.293, .570]). Simple effect analysis showed that when facing high levels of role changes (+1 *SD*), prompting a present- (vs. past-) focus coping benefits chronically mindful people (+1 *SD*: *b* = -1.49, *SE* = .36, *t*(422) = -4.15, *p* < .001, 95% CI = [-2.189, .783]), but not chronically unmindful people (*b* = -.01, *SE* = .37,*t*(422) = -.04, *p* = .968, 95% CI = [-.744, .714]). No effect of present vs. past focus emerged when facing little role change.

***Avoidant coping*.** Avoidant coping includes behaviors such as denial, venting, and behavioral disengagement [5] and it has been shown to correlate with self-alienation and inauthentic behaviors [6]. When people focus on the present, they should be less likely to avoid dealing with changes in their roles. Hence, we tested the possibility that present focus helped people cope with inauthenticity because it reduced avoidant coping.

We regressed avoidant coping on role change, past-focus, future-focus, role change X past-focus, role change X future-focus, and valence of change. Results yielded interactions of past-focus X role change (*β* = .18, *t*(428) = 3.41, *p* < .001, 95% CI = [.099, .369]) and future-focus X role change (*β* = .15, *t*(428) = 2.71, *p* = .007, 95% CI = [.051, .318]), main effects of past-focus (*β* = .08, *t*(428) = 1.90, *p* = .058, 95% CI = [-.004, .261]), future-focus (*β* = .08, *t*(428) = 1.78, *p* = .075, 95% CI = [-.012, .253]), role change (*β* = .38, *t*(428) = 5.47, *p* < .001, 95% CI = [.180, .382]), and valence (*β* = .20, *t*(428) = 5.22, *p* < .001, 95% CI = [.092, .202]).

Next, we tested avoidant coping as a mediator of the observed coping effect of present (vs. past and future) focus on inauthenticity. Using SPSS PROCESS Model 8, we conducted a moderated mediation analysis, while using inauthenticity as DV, role change as IV, temporal focus as moderator (as before, present focus was the benchmark), and valence of change as covariate (PROCESS Model 8). Results yielded a moderated mediation (bootstrapped sample = 5000, past- vs. present-focus moderator: 95% CI = [.147, .763]; future- vs. present-focus moderator: 95% CI = [.069, .650]). To test robustness, however, a second model testing the reversed direction between avoidant coping and inauthenticity was conducted. It also yielded a significant moderated mediation (past- vs. present-focus moderator: 95% CI = [.015, .240]; future- vs. present-focus moderator: 95% CI = [.022, .242]), suggesting that avoidant coping might be a consequence, rather than a mediator, of inauthenticity. Taken together, our results raised the possibility that inauthenticity and avoidant coping might have *bidirectional* effect.

# References to appendices

1. Rosenberg M. Society and the Adolescent Self-Image. Princeton: Princeton University Press; 1965.

2. Cameron JE. A Three-Factor Model of Social Identity. Self Identity. 2004;3(3):239-262. doi:10.1080/13576500444000047

3. Wood AM, Linley PA, Maltby J, Baliousis M, Joseph S. The authentic personality: A theoretical and empirical conceptualization and the development of the Authenticity Scale. J Couns Psychol. 2008;55(3):385-399. doi:10.1037/0022-0167.55.3.385

4. Feldman G, Hayes A, Kumar S, Greeson J, Laurenceau J. Mindfulness and Emotion Regulation: The Development and Initial Validation of the Cognitive and Affective Mindfulness Scale-Revised (CAMS-R). J Psychopathol Behav Assess. 2006;29(3):177-190.doi:10.1007/s10862-006-9035-8

5. Eisenberg SA, Shen BJ, Schwarz ER, Mallon S. Avoidant coping moderates the association between anxiety and patient-rated physical functioning in heart failure patients. J Behav Med. 2011;35(3):253-261. doi:10.1007/s10865-011-9358-0

6. Pinto D, Maltby J, Wood A, Day L. A behavioral test of Horney’s linkage between authenticity and aggression: People living authentically are less-likely to respond aggressively in unfair situations. Pers Individ Dif. 2012;52(1):41-44. doi:10.1016/j.paid.2011.08.023
